# Plaque disclosing agent as a plaque control guide for oral hygiene in chronic periodontitis based on guided biofilm therapy: A retrospective cohort study

**DOI:** 10.1097/MD.0000000000042782

**Published:** 2025-06-06

**Authors:** Bing Lei, Jin Liu, Shanmei Zhao, Cheng Chen, Zheng Cheng, Tianhua Yao, Limei Zhang, Xubing Zhao

**Affiliations:** a Key Laboratory of Shaanxi Province for Craniofacial Precision Medicine Research, College of Stomatology, Xi’an Jiaotong University, Xi’an, China; b Department of General Dentistry, College of Stomatology, Xi’an Jiaotong University, Xi’an, China; c Department of Periodontology, College of Stomatology, Xi’an Jiaotong University, Xi’an, China.

**Keywords:** guided biofilm therapy, oral hygiene guidance, periodontitis, plaque disclosing agent

## Abstract

We assessed the effectiveness of plaque disclosing agents as a visual aid for biofilm removal during professional oral hygiene instruction. A total of 220 patients with chronic periodontitis were enrolled in the study and divided into a control group (CG; traditional interventions) and an observation group (OG; guided biofilm therapy concepts applied). Plaque index (PI), bleeding on probing (BOP), and pocket depth (PD) were compared between the 2 groups. Self-care efficacy scale scores were assessed and compared, and oral hygiene behaviors were evaluated. After a 3-month intervention, the PI, BOP, and PD in OG reduced compared with those in CG. The total scores of the self-care efficacy scale in OG were higher than those in CG. The application of guided biofilm therapy concepts in the treatment and maintenance of patients with chronic periodontitis proves effective in reducing PI, BOP, and PD levels, enhancing patients’ oral health management abilities, and ensuring sustained therapeutic outcomes.

## 1. Introduction

Chronic periodontitis (CP) refers to a common oral disease that occurs in the periodontal support tissue. Approximately 12.5% of adults worldwide experience severe periodontitis.^[1]^ The estimated prevalence of this disease in the United States exceeds 50%.^[2]^ The results of the fourth national oral health epidemiological survey in China show that the periodontal health rate of the adult population is declining, and CP is the main cause of tooth loss, especially multiple tooth loss, in middle-aged and elderly people.^[3]^ Tooth loss can affect a person’s chewing ability and may have a negative effect on nutrition. The main pathogenic factor of CP is dental plaque that is generated by adhesion and coaggregation with mineral salts in saliva and gingival crevicular, that is, mineralized biofilm.^[4,5]^ Mineralized biofilm stimulates the gums, causing redness, swelling, and bleeding.^[6]^ Plaque is the initiating factor of CP,^[7]^ and even after removal, it could continue to form on the tooth surface. Therefore, plaque control is not confined to a specific stage of treatment but is integrated throughout the entire therapeutic process of CP. Plaque control is a necessary measure for the treatment and prevention of CP. Especially after treatment, plaque control is a key method to improve the prognosis of CP.^[8]^

Dental plaque biofilm accumulates in hard-to-reach areas where its removal becomes problematic, subsequently impairing effective home-based oral hygiene practices. The core content of the CP treatment plan is plaque control.^[9,10]^ Targeted removal of plaque, optimization of oral hygiene guidance (OHG), and professional tooth cleaning based on plaque conditions can help control the biofilm of whole mouth plaque.^[10]^ The control of plaque after systemic periodontal treatment is an important guarantee for the treatment effect of CP.^[11]^ Patients must learn the methods of plaque control at home^[12]^ and implement them lifelong after treatment to ensure the progress of periodontal treatment and maintain long-term efficacy.

Controlling biofilm through frequent mechanical removal plays a core role in the nonsurgical treatment of periodontitis.^[8]^ However, most guided biofilm therapy (GBT) protocol studies were performed during basic periodontal treatment. Plaque testing is typically performed in hospital settings rather than outpatient facilities. Few reports have been conducted on the application of this method in providing guidance on home oral hygiene for patients. As a basic principle, CP can be improved by personal oral hygiene measures. Therefore, on the basis of GBT protocol, this study aimed to guide patients to improve oral hygiene maintenance methods through self-testing of plaque. Based on the plaque display results, the plaque was effectively eliminated, thus improving the treatment effect of CP. The advantages of the GBT protocol were revealed by comparing it with the traditional periodontal disease treatment strategies, providing a clinical theoretical basis for OHG.

## 2. Research population and methods

The case-control studies of reporting guidelines have been used.

### 2.1. Study population

A total of 220 patients with CP from the College of Stomatology, Xi’an Jiaotong University, from October 2020 to October 2022, were selected as the research subjects. The patients were divided into a control group (CG) and an observation group (OG) in accordance with whether they adopted traditional interventions or interventions based on the concept of GBT applied to OHG. CG received the traditional intervention, whereas OG received GBT-based OHG.

Inclusion criteria are given as follows: met the diagnostic criteria for CP,^[13]^ a total of ≥15 teeth (excluding implants, crowns, and various dentures), underwent periodontal basic treatment, and voluntary receiving of OHG and follow-up.

Exclusion criteria are given as follows: age < 18 years; cognitive, communication, or intellectual impairment or unclear language expression; severe gingival hyperplasia; pregnant or lactating women; and severe hand diseases.

This study was conducted with the approval of the Ethics Committee of the Hospital of Stomatology (xjkqll[2019] No. 023), Xi’an Jiaotong University, and informed consent was obtained from all participants.

### 2.2. Methodology

The patients in both groups followed the 8-step treatment process of GBT, briefly described as follows.

1. Probing: Before treatment, a full-mouth examination was conducted to assess the condition of each tooth, including a comprehensive diagnosis of periodontal pocket depth (PD), gingival bleeding, gingival recession, and furcation involvement. An individualized treatment plan suitable for each tooth was formulated on the basis of assessment results.
2. Plaque disclosing agent staining: Plaque disclosing agent (Guilin Feist Medical Equipment Co Ltd, Guilin, China) is a dental material made from plant or fruit. It can stain plaque on the surface of teeth into easily recognizable colors. By observing the staining, the location and severity of plaque accumulation were judged.
3. OHG: Based on plaque staining, medical staff provides OHG to patients on their existing oral problems, emphasizing the importance of oral prevention and guiding them to master correct oral hygiene measures, including the following points: toothbrush selection: small-headed soft-bristled toothbrush should be chosen and replaced every 3 months; choosing toothpaste in accordance with the doctor’s advice; correct brushing methods: circular brushing, vertical brushing, and bass brushing (the most effective brushing method) were suggested; brushing time: brushing 2 times a day, with brushing before sleeping being particularly important, and adding an additional brushing after lunch is recommended; each brushing should last at least 3 to 5 minutes; and demonstration of the correct use of dental floss and interdental brushes: if patients have electric water flossers or electric toothbrushes, they should be taught how to use them.
4. Supragingival air polishing: Airflow, a sandblasting technology with glycine particles, is used as the main component for treatment.
5. Subgingival air polishing: Perio flow, a subgingival ultrasonic air-polishing technology, is used for treatment beneath the gums.
6. Painless ultrasonic scaling: Painless ultrasonic technology is used for scaling.
7. Evaluation of the effectiveness of scaling.
8. Scheduling the next visit: The next treatment appointment is booked in accordance with the treatment plan.

Regarding oral hygiene education and guidance in the third step, the content that patients need to grasp is the same, but the methods used for education and guidance differ between CG and OG.

### 2.3. Oral hygiene education and OHG in CG

After the treatment, routine chair-side oral health education was conducted. Oral hygiene maintenance methods on how to use a toothbrush, dental floss, or interdental brush were demonstrated on the model. The basic content that the patients need to master was explained.

### 2.4. Oral hygiene education and OHG in OG

**Figure 1.**
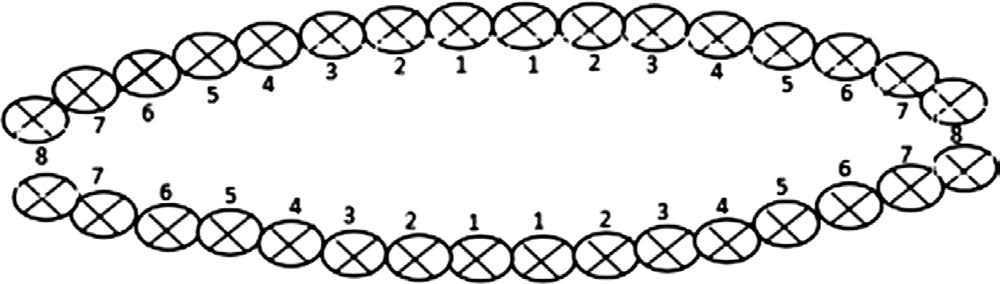
Plaque control record card.^[14]^

According to the second step of GBT, plaque disclosing agent was used for staining, and a plaque control record card (Fig. 1) was used for plaque marking. The patient’s missing teeth, implanted teeth, or fixed dentures are marked with “/.” Removable dentures were taken off. The teeth surfaces with plaque are marked with “x.” Plaque index (PI) was calculated. The patients were informed of the plaque situation. They were asked to self-examine the stained plaque areas in front of the mirror. Then, health education based on the patients’ existing oral problems was conducted for them to fully understand the importance of oral hygiene and the necessity of plaque removal. A toothbrush and toothpaste were provided to all patients. Dental floss or interdental brush was distributed in accordance with the patients’ tooth gap situation.

#### 2.4.1. Toothbrushing

Patients brush their teeth on site, while medical staff assist in recording toothbrushing duration and observe the brushing techniques. After brushing, the medical staff inspected the brushing effect and informed the patient about the remaining plaque. Correct brushing techniques are explained, and incorrect methods are pointed out. The patient is taught to clean all surfaces of the teeth, including the occlusal, labial, buccal, palatal, and lingual surfaces, until all plaque is removed. Patients are informed about the importance of brushing in a certain order and the necessary duration, ensuring that no areas are missed. They were also taught how to use plaque staining agents and observe the stained areas.

#### 2.4.2. Proximal cleaning

The size of the dental floss or interdental brush was selected on the basis of the size of the patients’ dental gaps. The patients were taught how to choose suitable proximal cleaning tools. With a mirror in one hand, the patients were shown how to place the proximal cleaning tool and effectively remove plaque. Especially for patients with exposed tooth roots, cleaning these areas thoroughly was crucial. The goal was to remove all plaques from the proximal surfaces of each tooth.

#### 2.4.3. Other OHG

The patients were asked to choose a small-headed, soft-bristled toothbrush and replace it every 3 months. If the patients used an electric toothbrush or water flosser, they were informed of the usage methods and precautions. Bringing the electric toothbrush or water flosser to the hospital for on-site guidance was preferable.

#### 2.4.4. Home oral hygiene cleaning method

The method started with plaque staining, followed by brushing in accordance with the staining results and then cleaning the proximal surfaces, observing the remaining plaque, and continuing to clean the plaque in those areas. When performing oral hygiene, the instructions provided must be followed, and attention must be paid to the precautions and key points of the operation as much as possible.

#### 2.4.5. Continuity of care

Patients agreed to join a WeChat group, where periodontal knowledge and videos were shared every 2 weeks. Monthly telephone follow-ups were conducted to understand the patients’ oral hygiene implementation, address any difficult issues in a timely manner, and provide guidance for any oral hygiene problems encountered by the patients. The intervention was continued for 3 months.

### 2.5. Observation indicators

#### 2.5.1. Questionnaire survey on oral hygiene behavior

General questionnaire: A self-designed questionnaire was established by the researcher, referencing the periodontal disease questionnaire in the “Fourth National Oral Health Epidemiological Survey Report.”^[3]^ The questionnaire included 2 parts: a general survey and an oral hygiene maintenance survey. The general situation included basic demographic characteristics, such as name, gender, and age. The disease-related surveys included tooth loss, periodontal disease classification, and types of toothbrushes used. The oral hygiene maintenance survey included “frequency of brushing teeth per day, duration of each brushing, brushing method, daily use of dental floss or interdental brush, and brushing time,” with a total of 5 items. After reliability analysis was conducted, the Cronbach α coefficient of the questionnaire reached 0.915, indicating good reliability.

#### 2.5.2. Self-efficacy scale for self-care on self-efficacy

Developed by Kakudate et al,^[15,16]^ the self-efficacy scale for self-care (SESS; Table 1) is used to assess the self-efficacy of oral health care. After being translated into Chinese, the SESS contained 3 dimensions and 15 items. Each item was rated using a Likert 5-point scale, ranging from “very confident” to “not confident at all,” with scores ranging from 5 points to 1 point. The total score ranged from 75 to 15 points, and a higher score indicates better self-oral health care by the patient.

**Table 1:** Self-efficacy scale for self-care questionnaire.

Items	
A. SE-DC (5 items)	B. SE-B (5 items)
I go to the dentist for treatment of periodontal disease.	I brush my teeth as instructed.
I cooperate with my dentist and hygienist for the treatment of periodontal disease.	I brush my teeth carefully and thoroughly.
I visit my dentist regularly, even after treatment is completed, to prevent recurrence.	I brush the border between the teeth and gums.
I have regular checkups even when I am busy with work or housework.	I move the toothbrush with a short, quick motion.
I have regular checkups even when my mind is not relaxed.	I take time to brush my teeth carefully.
C. SE-DH (5 items)	
I try not to spend too much time eating.	
I eat my meals at fixed times during the day.	
I try to eat a well-balanced diet.	
I try not to drink right before bed.	
I try not to eat too many sweets.	

SE-B = self-efficacy for brushing of the teeth, SE-DC = self-efficacy for dentist consultations, SE-DH = self-efficacy for dietary habits.

All questionnaire surveys were conducted 3 months after treatment.

#### 2.5.3. Plaque index

Plaque assessment was conducted using the O’Leary Plaque Control Record Card^[14]^ (Fig. 1), which is a widely used international evaluation method that helps patients record the effectiveness of plaque control. PI was calculated as the total number of teeth surface with plaque/total number of teeth surface examined ×100%, where the total number of teeth surface examined = total number of teeth × 4. The lower the PI, the better the oral hygiene status. The assessment was conducted during the 3-month follow-up after treatment.

#### 2.5.4. Bleeding on probing and PD

The percentages of bleeding on probing (BOP) and PD with a depth of ≥4 mm (PD4) were evaluated. The buccal and lingual sides of each tooth have 3 sides: mesial, middle, and distal, totaling 6 sites. The total number of sites = total number of teeth × 6. For each site: 0 = no BOP, 1 = BOP; 0 = periodontal PD < 4 mm, and 1 = periodontal PD ≥ 4 mm. The BOP percentage was calculated as the number of bleeding sites/total number of sites × 100%, and the PD4 percentage was calculated as the number of sites with periodontal PD ≥ 4 mm/total number of sites × 100%. The assessment was conducted during the 3-month follow-up after treatment.

#### 2.5.5. Methods of data collection

The assessment, probing, plaque detection, and treatment of teeth were performed by 2 experienced periodontists. The questionnaire survey, teaching oral hygiene maintenance methods to patients, online video push, and telephone follow-up were conducted by 4 experienced nurses. Doctors and nurses underwent unified training. Clinical data were collected after strict screening and passing the assessment.

#### 2.5.6. Statistical methods

A database was established using Epidata, with double entry by 2 individuals to control for entry errors. SPSS (version 20.0; SPSS Inc., Chicago) was used for data analysis. Numerical variables were expressed as the mean ± standard deviation. Categorical variables were expressed as frequency and percentage. Independent sample *t* tests were used for comparisons of measurement data. The χ^2^ tests were used for comparisons of categorical data. A *P* value <0.05 was considered statistically significant.

## 3. Results

### 3.1. Patients

**Figure 2.**
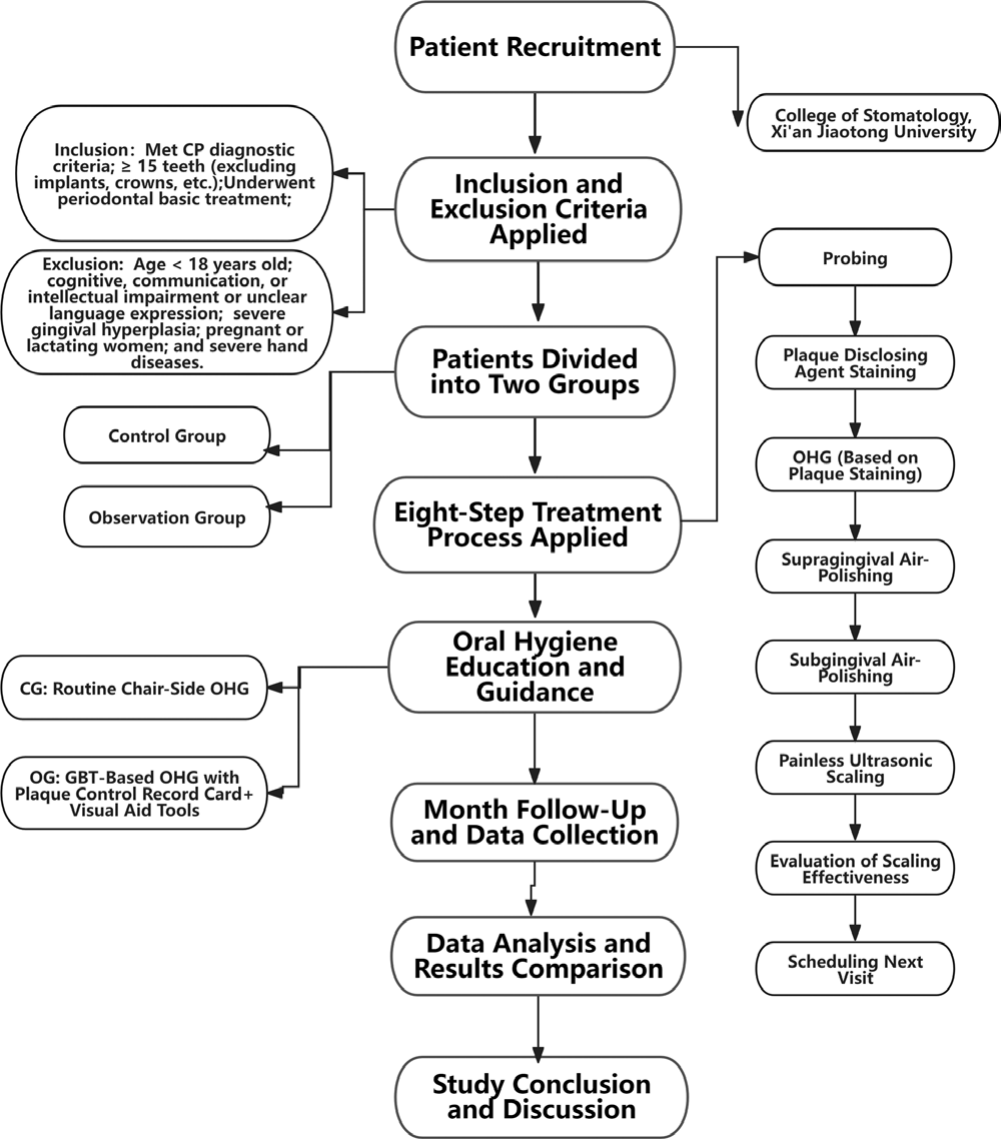
Flowchart showing the recruitment process and follow-up of patients. CG = control group, CP = chronic periodontitis, GBT = guided biofilm therapy, OG = observation group, OHG = oral hygiene guidance.

A total of 220 patients with CP were included in the study, among which 15 patients were lost to follow-up. Finally, 103 patients in CG and 102 patients in OG were included (Fig. 2). No statistically significant differences were found between the 2 groups in terms of gender, age, educational level, occupation, and income (*P* > .05).

### 3.2. Comparison of plaque detection within OG before and after guidance

**Figure 3.**
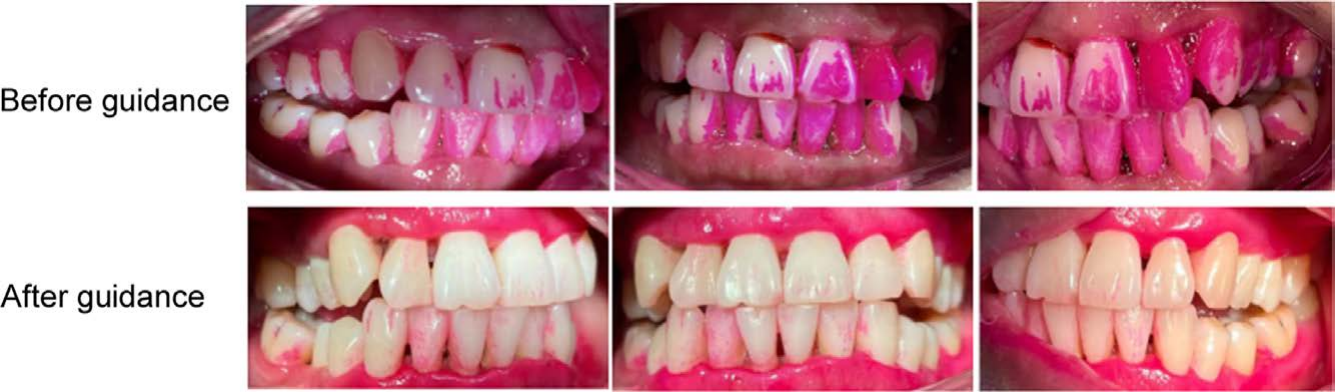
Comparison of plaque detection before and after guidance.

Under OHG based on GBT, the effectiveness of biofilm removal in the OG group was significantly improved (Fig. 3).

### 3.3. Comparison of oral hygiene behavior

Before the intervention, no statistically significant difference was found in each item between CG and OG, with *P *> .05. After the intervention, OG was superior to CG in terms of daily brushing frequency, duration of each brushing, use of dental floss or interdental brushes, and brushing methods. A statistically significant difference was observed in each item between the 2 groups (*P *< .001; Table 2).

**Table 2:** Comparison of oral hygiene behaviors between 2 groups of patients before and after intervention (control group n = 103; observation group n = 102).

Item	Group	Before treatment, n (%)		χ^2^	*P* value	3 months, n (%)		χ^2^	*P* value
		Control	Observation			Control	Observation		
Daily brushing frequency	1	24 (23.30)	19 (18.63)	0.772	.680	3 (2.91)	1 (0.98)	6.541	.038
	2	68 (66.02)	70 (68.63)			75 (72.82)	60 (58.82)		
	≥3	11 (10.68)	13 (12.75)			25 (24.27)	41 (40.20)		
Duration of brushing	1 min	45 (43.69)	40 (39.22)	0.551	.759	16 (16.36)	1 (0.98)	79.518	<.001
	2 min	50 (48.54)	52 (50.98)			53 (38.18)	6 (5.88)		
	≥3 min	8 (7.77)	10 (9.80)			34 (45.45)	95 (93.14)		
Whether to use dental floss or interdental brush	Yes	9 (8.74)	13 (12.75)	1.854	.396	49 (47.57)	74 (72.55)	29.808	<.001
	No	83 (80.58)	74 (72.55)			46 (44.66)	11 (10.78)		
	Occasional	11 (10.68)	15 (14.71)			8 (7.77)	17 (16.67)		
Brushing methods	Correct	11 (10.68)	13 (12.75)	0.212	.646	34 (33.01)	84 (86.27)	51.079	<.001
	Incorrect	92 (89.32)	89 (87.25)			69 (66.99)	18 (17.65)		

### 3.4. Comparison of SESS scores

The patients were evaluated using the SESS at 3 months after intervention. The differences in self-efficacy scores for dentist consultations, correct brushing, and balanced eating were statistically significant between the 2 groups (*P *< .001; Table 3).

**Table 3:** Comparison of self-efficacy scale for self-care scores (x ± s) between 2 groups of patients.

Group	n	Self-efficacy scores for dentist consultations		Self-efficacy scores for correct brushing		Self-efficacy scores for balanced eating	
		Before treatment	3 months	Before treatment	3 months	Before treatment	3 months
Control	103	10.32 ± 3.12	14.47 ± 4.17	7.67 ± 3.69	13.33 ± 5.26	11.77 ± 3.18	14.56 ± 3.78
Observation	100	10.37 ± 3.12	23.25 ± 14.47	7.79 ± 3.59	23.76 ± 1.97	11.83 ± 2.74	23.54 ± 1.37
t value		-0.104	19.670	0.244	18.794	0.160	22.548
*P* value		.917	<.001	.807	<.001	.873	<.001

### 3.5. Comparison of PI, BOP, and PD ≥ 4

Before the intervention, no statistically significant differences were observed in the PI, BOP, and PD ≥ 4 between the 2 groups (*P* > .05). Three months after the intervention, statistically significant differences were found in PI (*P *< .001), BOP (*P* < .001), and PD ≥ 4 (*P* = .002) between the 2 groups (Table 4).

**Table 4:** Comparison of clinical indicators between 2 groups of patients.

Group	n	PI ($\bar{x}\pm \;s$)		BOP + % ($\bar{x}\pm \;s$)		PD4 + % ($\bar{x}\pm \;s$)	
		Before treatment	3 months	Before treatment	3 months	Before treatment	3 months
Control	103	66.91 ± 16.46	44.27 ± 15.74	44.63 ± 22.49	28.76 ± 16.12	42.54 ± 21.28	26.12 ± 14.91
Observation	102	66.18 ± 18.52	31.80 ± 14.06	47.99 ± 24.14	23.39 ± 18.53	41.93 ± 23.08	19.18 ± 16.56
*t* value		−0.299	−5.980	−0.829	−10.001	−1.969	−3.157
*P* value		.765	<.001	.408	<.001	.845	.002

BOP = bleeding on probing, PD = pocket depth, PI = plaque index.

## 4. Discussion

### 4.1. Controlling plaque via GBT: the cornerstone of CP management

Given CP’s irreversible nature, mitigating its progression forms the cornerstone of treatment. Self-care maintenance, encompassing dental plaque control and regular follow-ups, is vital to prevent periodontitis recurrence.^[17]^ Post-treatment, adherence to plaque control, regular scaling, and gum health monitoring ensures long-term efficacy. Supragingival plaque management is pivotal for sustaining outcomes and averting relapses.^[18]^ Plaque disclosing agents facilitate visual monitoring, enabling prompt adjustments to prevention strategies, and enhancing plaque removal efficiency.^[19]^ Following systematic therapy, most patients with CP experience inflammation resolution; however, daily plaque control remains essential during maintenance. PD < 4 mm signifies periodontal health, whereas BOP presence signals ongoing inflammation.^[20]^ In this study, GBT-guided plaque control was applied to OHG. The 3-month intervention showed a significant decrease in PI, BOP, and PD. The patients in OG applied the GBT concept to OHG, which allowed for feedback on the reasons for poor plaque control behavior and timely correction of improper oral care methods. Adjusting the content of plaque control management can avoid untargeted health education. This retrospective study suggests that plaque control based on the GBT concept is the key to controlling CP.

### 4.2. Enhancing patient self-management and efficacy through GBT-based OHG

Patients with CP often struggle with oral hygiene due to knowledge gaps, incorrect techniques, and ingrained poor habits. Post-treatment, inadequate care exacerbates poor outcomes. GBT in OHG addresses individual hygiene habits and disease trajectories, offering personalized education tailored to patient needs. This approach enhances effective healthcare provider-patient interactions, empowering patients with accurate knowledge and techniques. Our study reveals higher SESS scores in the GBT group, attesting to improved self-management, which is consistent with the study by Džiaugytė et al.^[21]^ Focusing on doctor’s advice, brushing techniques, and balanced diets advances long-term periodontal health. The application of the GBT concept in OHG resulted in enhanced communication between healthcare providers and patients, thus eliminating negative emotions and gaining the trust of patients, ultimately achieving the goal of improving self-management abilities.

### 4.3. Fostering positive self-care behaviors via GBT-oriented OHG

While brushing removes plaque, mineralized biofilm necessitates professional removal. GBT-guided OHG nurtures proper hygiene knowledge and habits, curbing plaque regrowth. Effective brushing techniques^[22]^ and regular interproximal cleaning^[23]^ are cornerstone strategies. In this study, OG had a higher proportion of patients who brushed their teeth at least 2× a day, brushed for at least 3 minutes each time, used dental floss or interproximal brushes daily, and adopted correct brushing techniques than CG. Our study shows that GBT application increased daily brushing frequency, duration, and use of dental floss/brushes, indicating behavioral shifts toward proactive oral care. This not only elevates oral health awareness but also equips patients with CP prevention and self-care skills, ultimately contributing to better CP management.

## 5. Conclusion

GBT-integrated OHG significantly diminishes PI, BOP, and PD, demonstrating its clinical efficacy. It also elevates the oral hygiene self-management of patients with CP, heightening oral health consciousness. GBT-based periodontal treatments effectively curb disease progression, safeguard teeth, and improve patients’ quality of life.

## Author contributions

**Conceptualization:** Xubing Zhao, Bing Lei.

**Writing – original draft:** Xubing Zhao, Bing Lei.

**Investigation:** Bing Lei, Jin Liu, Shanmei Zhao, Cheng Chen, Zheng Cheng, Tianhua Yao, Limei Zhang.

**Methodology:** Bing Lei.
